# Gene Expression Programs in Response to Hypoxia: Cell Type Specificity and Prognostic Significance in Human Cancers

**DOI:** 10.1371/journal.pmed.0030047

**Published:** 2006-01-24

**Authors:** Jen-Tsan Chi, Zhen Wang, Dimitry S. A Nuyten, Edwin H Rodriguez, Marci E Schaner, Ali Salim, Yun Wang, Gunnar B Kristensen, Åslaug Helland, Anne-Lise Børresen-Dale, Amato Giaccia, Michael T Longaker, Trevor Hastie, George P Yang, Marc J van de Vijver, Patrick O Brown

**Affiliations:** 1Department of Biochemistry, Stanford University School of Medicine, Palo Alto, California, United States of America; **2**The Institute of Genome Sciences and Policy, Department of Molecular Genetics and Microbiology, Duke University School of Medicine, Durham, North Carolina, United States of America; **3**Department of Surgery, Stanford University School of Medicine, Palo Alto, California, United States of America; **4**Diagnostic Radiation Oncology, The Netherlands Cancer Institute, Amsterdam, The Netherlands; **5**Department of Radiation Oncology, Stanford University School of Medicine, Palo Alto, California, United States of America; **6**Department of Gynecologic Oncology, The Norwegian Radium Hospital, Oslo, Norway; **7**Department of Genetics, The Norwegian Radium Hospital, Oslo, Norway; **8**Health Research and Policy, Stanford University School of Medicine, Palo Alto, California, United States of America; **9**Palo Alto VA Health Care System, Palo Alto, Stanford University School of Medicine, Palo Alto, California, United States of America; **10**Howard Hughes Medical Institute, Stanford University School of Medicine, Palo Alto, California, United States of America; Genome Institute of SingaporeSingapore

## Abstract

**Background:**

Inadequate oxygen (hypoxia) triggers a multifaceted cellular response that has important roles in normal physiology and in many human diseases. A transcription factor, hypoxia-inducible factor (HIF), plays a central role in the hypoxia response; its activity is regulated by the oxygen-dependent degradation of the HIF-1α protein. Despite the ubiquity and importance of hypoxia responses, little is known about the variation in the global transcriptional response to hypoxia among different cell types or how this variation might relate to tissue- and cell-specific diseases.

**Methods and Findings:**

We analyzed the temporal changes in global transcript levels in response to hypoxia in primary renal proximal tubule epithelial cells, breast epithelial cells, smooth muscle cells, and endothelial cells with DNA microarrays. The extent of the transcriptional response to hypoxia was greatest in the renal tubule cells. This heightened response was associated with a uniquely high level of HIF-1α RNA in renal cells, and it could be diminished by reducing HIF-1α expression via RNA interference. A gene-expression signature of the hypoxia response, derived from our studies of cultured mammary and renal tubular epithelial cells, showed coordinated variation in several human cancers, and was a strong predictor of clinical outcomes in breast and ovarian cancers. In an analysis of a large, published gene-expression dataset from breast cancers, we found that the prognostic information in the hypoxia signature was virtually independent of that provided by the previously reported wound signature and more predictive of outcomes than any of the clinical parameters in current use.

**Conclusions:**

The transcriptional response to hypoxia varies among human cells. Some of this variation is traceable to variation in expression of the *HIF1A* gene. A gene-expression signature of the cellular response to hypoxia is associated with a significantly poorer prognosis in breast and ovarian cancer.

## Abstract 2

The transcriptional response to hypoxia varies between cell types. A gene-expression signature of the cellular response to hypoxia is associated with a significantly poorer prognosis in breast and ovarian cancer.

## Introduction

Oxygen is essential for aerobic metabolism in all mammalian cells. To maintain function and homeostasis, cells have to be able to sense and respond to inadequate oxygen levels (hypoxia). A family of transcription factors called hypoxia-inducible factors (HIFs) plays a central role in orchestrating the cellular response to hypoxia. HIF-1, the first member of the family, is a heterodimeric protein consisting of a constitutively expressed subunit, HIF-1β, and an oxygen-sensitive inducible subunit, HIF-1α. HIF-1α protein is usually degraded under normal oxygen concentrations (normoxia), but in hypoxic conditions or in the presence of iron chelators, the degradation rate decreases, and HIF-1α protein accumulates and associates with HIF-1β to form a functional transcription complex, triggering the transcription of a host of hypoxia-inducible genes. Recent studies have shed light on the mechanisms of hypoxia-driven stabilization of HIF-1α protein. Under normal oxygen tension, HIF-1α protein is hydroxylated on two proline residues by a family of oxygen-dependent prolyl hydroxylases (PHD1–3), and the modified HIF-1α becomes a substrate for polyubiquitination by a protein complex containing von Hippel-Lindau proteins (pVHL) and is thus targeted for degradation [1]. The enzymatic activities of PHD proteins are sensitive to oxygen availability. Under low-oxygen conditions, PHD proteins are unable to modify the HIF-1α protein, keeping HIF-1α protein unhydroxylated and allowing it to escape pVHL recognition and subsequent degradation, resulting in the triggering of the hypoxia responses [2].

HIF-1α protein stabilization, however, is not limited to hypoxic conditions, and the so-called hypoxia responses can occur even with an adequate oxygen supply. This oxygen tension-independent hypoxia response can result from a wide range of possible genetic alterations and signaling malfunctions, including loss of VHL [3], p53 [4], or PTEN (a phosphatidylinositol trisphosphate lipid phosphatase) [5]; or activation of phosphatidylinositol 3-kinase/Akt [6] or Src pathways [7]. Hypoxia-independent HIF-1α protein stabilization is seen in patients with von Hippel-Lindau disease, a genetic disease in which one copy of the *VHL* gene is either inactivated or deleted. In these patients, when the remaining normal copy of the *VHL* gene is lost or inactivated, the HIF transcriptional complexes stay constitutively active, even under normal oxygen concentrations, due to faulty, pVHL-dependent degradation pathways. This dysregulated hypoxia pathway is a key factor in the development of multiple neoplasms in patients with von Hippel-Lindau disease [8].

Although hypoxia responses are thought to be evolutionarily conserved in all mammalian cells [9,10], not all cells respond to hypoxia in an identical fashion during physiological and pathological adaptations. Different cells of the human body have diverse energy requirements, operate in different microenvironments, and are normally exposed to different ranges of oxygen concentration. For example, bronchial epithelial cells are usually exposed to ambient atmospheric levels of oxygen, while human chondrocytes are trapped in a low-oxygen, avascular cartilage matrix and rely on diffusion from adjacent tissues for oxygen. Cells also differ in sensitivity to hypoxia; some cells can survive indefinitely in anoxic environments; others die within minutes. Because of their variable baseline situations and responses to hypoxia, cells of different tissues react differently when their hypoxia pathways are dysregulated. In von Hippel-Lindau disease, loss of pVHL function frequently leads to tumors arising from renal epithelial cells [8]. The molecular basis for the preferential sensitivity of renal epithelial cells to the oncogenic effects of the mutation in pVHL is unknown.

Genomic tools, including DNA microarrays, have enabled the global gene expression programs of cells placed under hypoxic stress to be studied systematically [10–14]. Although the heterogeneity of the hypoxia response has been recognized, there has been no systematic analysis of the extent and nature of this heterogeneity. In this study, we used DNA microarrays to examine the gene expression program in response to hypoxia in several different primary cells. We found significant variation in the hypoxia responses among different cell types, and these variations were correlated to the varying composition of hypoxia regulators in the different cell types. The study of cell-specific responses to hypoxia could lead to a better understanding of the regulation of oxygen sensing and energy metabolism, and it may provide insights into the basis of the anatomical specificity of diseases and injuries in which hypoxia or the hypoxia response play a role.

Although hypoxia has been recognized as an important determinant of clinical outcomes in human cancers [15], it has been difficult to perform comprehensive and quantitative analyses to define tumor phenotypes based on hypoxia responses and to explore the relationship between tumor hypoxia and genetic changes or clinical parameters in human cancers. Several previous reports have identified links between cancer outcomes and the level of HIF-1α protein or the expression of one or two individual genes that are induced by hypoxia, such as *Carbonic Anhydrase IX* [16–20]. In our own previous studies, we found that gene expression signatures of cell proliferation [21] and a wound response program [22] obtained from well-defined cell culture settings could provide a basis for recognizing the corresponding programs in human cancer tissues. In the present study, we tested the idea that a gene expression “signature” of the hypoxia response, derived from our systematic analysis of the responses in cultured primary epithelial cells, might serve as a molecular gauge to assess the extent to which this physiological response is activated in different human cancers, providing a new perspective on previously reported systematic gene expression datasets. We also devised an analytic framework to explore the relationship between hypoxia signatures and other molecular and clinical features in determining the clinical behaviors and patient outcomes of several different human cancers.

## Methods

### Cell Culture

Human coronary artery endothelial cells (ECs), smooth muscle cells (SMCs), human mammalian epithelial cells (HMECs), and renal proximal tubule epithelial cells (RPTECs) were purchased from Cambrex (East Rutherford, New Jersey, United States), and cultured in specified medium as suggested by manufacturer. To create hypoxic conditions, 70%–80% confluent cells were placed in a tissue culture incubator with 2% O_2_ and 5% CO_2_ at 37 °C. To create anoxic conditions, 70%–80% confluent cells placed in an anaerobic chamber (Sheldon, Cornelius, Oregon, United States) with less than 0.02% O_2_ and 5% CO_2_ at 37 °C.

### HIF-1α Quantitative Real-Time PCR

Total RNA was purified with Absolutely RNA microprep kit (Stratagene, La Jolla, California, United States). Quantitative real-time PCR was performed on an ABI PRISM 7900H machine (Applied Biosystems, Foster City, California, United States) with TaqMan reagents (Applied Biosystems). Primer sequences used for HIF-1α were: 5′ primer, 5′-CTCACCCAACGAAAAATTACAGAA-3′; 3′ primer, 5′-ATTGAGTGCAGGGTCAGCACTAC-3′, and probe, FAM-CATTACCCACCGCTGAAACGCCAA-TAMRA. TaqMan β-actin detection reagents (Applied Biosystems) were used as an internal control for quantitation. Quantitative real-time PCR was performed at 95 °C for 10 min followed by 40 cycles of denaturing at 95 °C for 15 s and annealing/extending at 60 °C for 1 min.

### HIF-1α Immunoblotting

Cell lysates were prepared by adding RIPA buffer immediately after hypoxia or anoxia. Total protein was quantitated with the BCA Protein Assay Reagent Kit (Pierce, Rockford, Illinois, United States) and equal amounts of protein loaded in each lane of a 10% SDS-PAGE gel followed by transfer to a PVDF membrane (Bio-Rad, Hercules, California, United States) at 90 V for 2 h. After blocking with 5% nonfat milk for 1 h at room temperature, the membrane was incubated with monoclonal antibodies specific for HIF-1α (BD Biosciences, San Jose, California, United States) overnight at 4 °C. After washing, horseradish peroxidase-linked anti-mouse IgG (Amersham Biosciences, Piscataway, New Jersey, United States) was used as a secondary antibody, and incubated with the membrane for 45 min at room temperature. Signal was detected by ECL Western blotting detection reagents (Amersham Biosciences).

### HIF-1α Dicer RNA Interference

The following three sets of primers were used generate fragments for HIF-1α Dicer RNA interference (RNAi) and incorporated T7 promoter sequences: HIF-1α forward primer-1, 5′-GCGTAATACGACTCACTATAGGGACACTGGTGGCTCACTACC-3′; HIF-1α reverse primer-1, 5′-GCGTAATACGACTCACTATAGGGTCCAGGTTTAACAATTTCATAGGCC-3′; HIF-1α forward primer-2, 5′-GCGTAATACGACTCACTATAGGGATGGTTCTCACAGATGATGGTGAC-3′; HIF-1α reverse primer-2, 5′-GCGTAATACGACTCACTATAGGGTTGAGCGGCCTAAAAGTTCTTC-3′; HIF-1α forward primer-3, 5′-GCGTAATACGACTCACTATAGGGCCAAGAATTCTCAACCACAGTGC-3′; and HIF-1α reverse primer-3, 5′-GCGTAATACGACTCACTATAGGGTCGGAAGGACTAGGTGTCTGATC-3′.

Firefly luciferase (GL3) and Renilla luciferase (RL) were used as controls. Sequences used were designed based upon previous reports [23]. DNA fragments with T7 promoters were generated by PCR and subjected to in vitro transcription to produce dsRNAs (550 bp) using the MEGAscript in vitro transcription kit (Ambion, Austin, Texas, United States). dsRNAs were treated with DNase I, and incubated with r-Dicer at 37 °C in 250 mM NaCl, 30 mM HEPES (pH 8.0), 0.05 mM EDTA, 2.5 mM MgCl_2_ overnight. Dicer-treated small interfering RNA (d-siRNA) was purified using the Micro-to-Midi Total RNA Purification System (Invitrogen) and transfected into RPTECs by electroporation by using the Basic Nucleofector Kit for primary mammalian epithelial cells and Nucleofector Device according to the directions provided by the manufacturer (Amaxa Biosystems, Gaithersburg, Maryland, United States). 24 h after transfection, the cells were subjected to hypoxia and RNAs harvested 12 h later for analysis.

### cDNA Microarrays and Hybridization

We used human cDNA microarrays containing 42,000 elements that represent 27291 unique genes. Arrays were produced at the Stanford Functional Genomic Facility. The RNAs were purified by FastTrack (Invitrogen), and fluorescently labeled cDNAs were hybridized to the array in a two-color comparative format, with the experimental samples labeled with one fluorophore (Cy-5) and a reference pool of mRNA labeled with a second fluorophore (Cy-3) [24].

### Data Filtering and Analysis

Array images were scanned by using an Axon Scanner 4000B (Axon Instruments, Union City, California, United States), and image analysis was performed by using Genepix Pro version 3.0.6.89 (Axon Instruments). Data were expressed as the log2 ratio of fluorescence intensities of the sample and the reference, for each element on the array [24]. Data were filtered to exclude elements that did not have at least a 2.5-fold intensity/background ratio in at least 60% of the arrays. The time course data from each cell type under hypoxia (2%) and anoxia (0%) were normalized to the ambient air control harvested at the same time, and the subset of elements that varied from the baseline by at least 3-fold in at least four samples was selected for further analysis. The data were hierarchically clustered by using the cluster program [25], and displayed by using TreeView [1]. The changes in gene expression for each gene were evaluated at each time point through zero transformation [26] by subtracting log2(red/green) of the normoxia (∼21% O_2_) measurement from the corresponding log2(red/green) ratio at hypoxia (2% O_2_) or anoxia (0% O_2_) at the same time points. The resulting values represent log2(red hypoxia/red normoxia), or log2(red anoxia/red normoxia) and so on, and indicate changes associated with either hypoxia (2% O_2_) or anoxia (0% O_2_). To identify genes with changes only one of the four cell types, we used multi-class significance analysis of microarrays (SAM) [27] to analyze variations of gene expression after zero-transformation associated with ECs, SMCs, HMECs or RPTECs. The cell type-specific gene list was selected to have a false discovery rate (FDR) of 3.7 %, using 100 iterations. For detailed procedures and complete data, please see Figure S1 and Tables S1 and S2.

### Analysis of an Epithelial Hypoxia Signature in Human Cancers

The epithelial hypoxia signature gene list consists of 253 unique image clones on the cDNA Stanford array by selecting a gene cluster showing induction in the tested epithelial cells (HMECs and RPTECs). For gene expression analysis of renal cell carcinoma [28], breast cancers [29], and ovarian cancer, the expression value of these clones was extracted and genes were selected for further analysis for which the corresponding array elements had fluorescent hybridization signals at least 2.5-fold greater than the local background fluorescence. We further restricted our analysis to genes for which adequate data were obtained in at least 80% of experiments. The image clones in the epithelial hypoxia signature that satisfied all the above criteria were used to stratify tumors in different datasets based on their hypoxia response with hierarchical clustering. For the analysis of breast cancer samples of Netherlands Cancer Institute (NKI), 35 of 253 unique image clones could not be mapped to a Unigene cluster. The 218 remaining clones were mapped to 168 unique Unigene clusters. The 168 Unigene cluster represented 180 unique sequences on the Rosetta/NKI oligo array. Cross-checking gene names revealed 22 probes that could not confidently be contributed to genes in the original hypoxia signature. These were removed, resulting in 158 matched probes. These 158 were the matching probes to 123 unique Unigene clusters. In order to overcome possible overestimation of Unigene clones that were matched to more than one probe on the NKI array, the probes that were not uniquely match to one Unigene cluster were averaged. Genes were mean centered and clustered and visualized in TreeView. Based on genes highly expressed in the hypoxia response, two groups of patients were identified. These were called, respectively, high- and low-hypoxia response. Overall survival was defined by death from any cause. Distant metastasis-free survival was defined by a distant metastasis as a first recurrence event; data on all patients were censored on the date of the last follow-up visit, death from causes other than breast cancer, the recurrence of local or regional disease, or the development of a second primary cancer, including contralateral breast cancer.

Kaplan-Meier survival curves were compared by the Cox-Mantel log-rank test in Winstat for Excel (R. Fitch Software, Staufen, Germany). Multivariate analysis by the Cox proportional hazard method was performed using the software package SPSS 11.5 (SPSS, Chicago, Illinois, United States).

## Results

### Analysis of the Cell-Type Specificity of Hypoxia Responses

**Figure 1:**
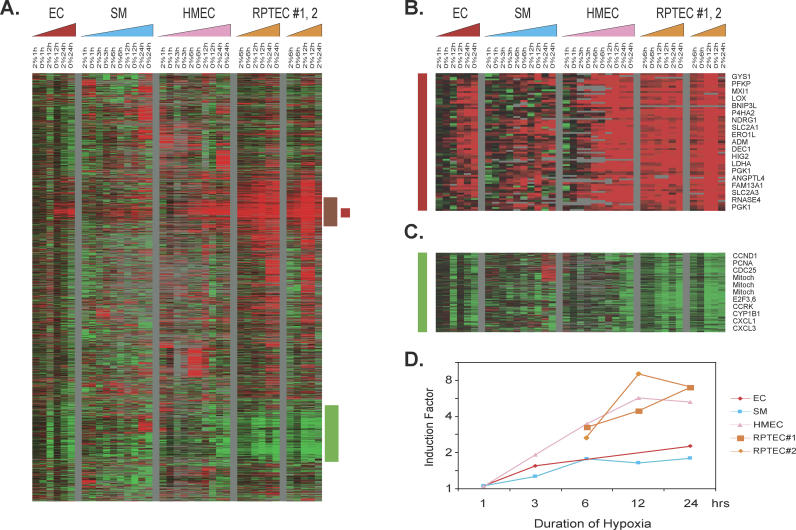
Overview of the Genomic Responses to Hypoxia (A–C) Hierarchical clustering of a total of 4,333 elements that display a greater than 3-fold change in mRNA expression in more than four different samples when exposed to hypoxia (A). Data from individual elements or genes are represented as single rows, and different time points in the time courses (triangles) are shown as columns. Red and green denote expression levels of samples cultured under hypoxia (2% O_2_) or anoxia (0% O_2_) greater or lower, respectively, than baseline values of samples cultured under ambient air (∼21% O_2_). The intensity of the color reflects the magnitude of the change from baseline. The color of the triangles represents the time course of the different cell types (red, ECs; blue, SMCs; pink, HMECs; orange, RPTECs). The vertical red bar marks a cluster of genes induced in all cells, termed the “common hypoxia genes” (B); the vertical brown bar marks a cluster of genes induced in all epithelial cells, termed the “epithelial hypoxia genes”; and the vertical green bar marks a cluster of genes repressed in all cells, termed the “commonly repressed hypoxia genes” (C). The gene clusters representing “common hypoxia genes” (B) and the “commonly repressed hypoxia genes” (C) are expanded to show the names of representative genes on the right side. (D) Average folds of gene induction (y-axis) in the common hypoxia genes cluster from each indicated cell type at different time points (x-axis) are calculated and shown. (Complete data can be found at: http://microarray-pubs.stanford.edu/hypoxia)

To characterize the gene expression program in response to hypoxia, and its variations among different cell types, we examined primary cells from several anatomic locations, including coronary artery ECs, coronary artery SMCs, HMECs, and two independent isolates of renal proximal tubular epithelial cells (RPTEC#1 and #2). These cells were expanded in culture and then exposed to culture environments of ambient oxygen level (∼ 21%), hypoxia (2%), and anoxia (0%) under identical CO_2_ concentrations (5%). We profiled global mRNA levels at five time points each for the coronary artery SMCs and HMECs (1, 3, 6, 12, and 24 h) and three time points each for coronary artery ECs (1, 3, and 24 h) and two independent cultures of RPTECs (6, 12, and 24 h). Different time points were chosen to characterize the temporal changes of gene expression patterns in the first 24 h of exposure to hypoxia. One of the cultured RPTEC samples (#2) suffered significant cell death after 24 h of anoxia and was not analyzed further. The 56 different mRNA samples were analyzed by hybridization to DNA microarrays, containing approximately 42,000 elements, representing 27,291 unique Unigene clusters (build number 173, released on 28 July 2004), to generate a total of 2.4 million gene expression measurements. Gene expression profiles of the hypoxia response of each cell type were derived by a zero transformation process [26] in which we compared transcript levels for each gene in cells cultured under low-oxygen conditions (2% and 0% O_2_) to the transcript levels in the same cell type (at the same time point) cultured in ambient air (∼21% O_2_). The 4,333 array elements, representing 3,725 Unigene clusters, which exhibited the largest hypoxia responses, are displayed in Figure 1A (the detailed expression values in different samples are available on the Stanford Microarray Database at: http://microarray-pubs.stanford.edu/hypoxia, with genes hierarchically clustered according to similarities in expression patterns.

There was significant heterogeneity in the cellular hypoxia responses among different cell types. We identified a cluster of “epithelial cell hypoxia genes” (highlighted by the vertical brown bar in Figure 1A) induced by hypoxia in all the tested epithelial cells and a cluster of “common hypoxia genes” (vertical red bar in Figure 1A; expanded in Figure 1B) induced by hypoxia in all tested cells. Another distinct set of genes is repressed in all cells (vertical green bar in Figure 1A; expanded in Figure 1C). The common hypoxia genes (Figure 1B) included many genes that have previously been associated with hypoxia responses, including genes encoding proteins with central roles in glucose transport (glucose transporters SLC2A1 and SLC2A3) and metabolism (phosphofructokinase [PFKP], glycogen synthase [GYS1], lactate dehydrogenase A [LDHA], and phosphoglycerate kinase [PGK1]), angiogenesis (angiopoietin 4 [ANGPTL4] and adrenomedullin [ADM]), differentiation (DEC1), cell proliferation and apoptosis (BNIP3L, NDRG1, and MXI1), extracellular matrix synthesis (prolyl 4-hydroxylase [P4HA1, P4HA2], and lysyl oxidase [LOX]), and other significant biological processes (HIG2, FAM13A1, and RNASE4). Although these genes were found to be hypoxia-inducible in all cell types in this and several previous studies (reviewed in [30]), significant variations were seen in the degree of induction among different cell types, as is evident from the intensity of signal in Figure 1B. The magnitude of induction of these common hypoxia genes was generally higher in the two primary epithelial cells (HMECs and both samples of RPTECs) than in the two samples of mesenchymal cells in this study (ECs and SMCs) (Figure 1D).

Among hypoxia-repressed genes were a large group of genes whose expression is closely linked to cell proliferation (cyclinD, PCNA, CCRK, E2F3, and E2F6) and a set of genes encoded by mitochondrial DNA, whose expression level may reflect variations in the number of mitochondria per cell. These hypoxia-repressed genes may reflect a physiological alteration that halts cell proliferation and translation activities as the cells try to preserve energy consumption under metabolic stress [31,32]. The mTOR pathways may be implicated in this cell cycle arrest brought about by hypoxia-associated energy depletion [33]. The mechanisms responsible for the regulation of these proliferation-linked mRNAs in response to hypoxia remain to be fully characterized; it is not clear whether the down-regulation of proliferative responses is directly mediated by sensing decreased O_2_ or indirectly due to alterations in the cells' energy economy.

**Figure 2:**
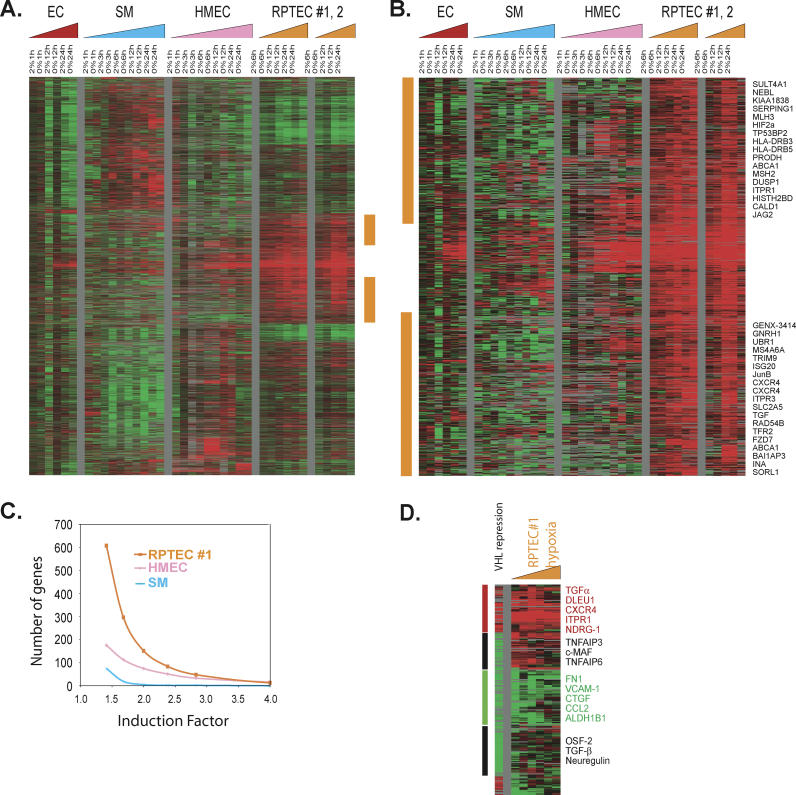
Cell Type–Specific Hypoxia Response (A and B) Hierarchical clustering of a total of 5,432 array elements showing a cell type-specific hypoxia response that are selected by multiclass SAM using zero-transformed gene expression with a FDR of 3.7% (A). The orange vertical bar marks clusters of genes induced only in RPTECs and is expanded with the names of representative genes (B). (C) The number of genes (y-axis) with fold inductions greater than those indicated (x-axis) in all six hypoxic samples (6, 12, and 24 h at 2% and 0% O_2_) are shown for SMCs (SM, blue curve), HMECs (pink curve), and RPTEC#1 (orange curve). (D) Comparison of the gene expression pattern of VHL-reconstituted RCCs (A498 cells) to the hypoxia response of RPTEC#1. We compared the expression of genes affected by VHL reconstitution of VHL-deficient RCC A498 cells (from [38] and in the sample marked “VHL repression”) to the expression of those same genes in RPTEC#1 with hypoxia treatments (samples marked “RPTEC#1 hypoxia”). Genes showing concurrent induction (vertical red bar), concurrent repression (vertical green bar), and VHL-specific response (vertical black bar) are shown with selected gene names. (Complete data can be found at: http://microarray-pubs.stanford.edu/hypoxia)

To identify cell type-specific hypoxia responses associated with each of the four different cell types, we used a permutation-based technique, SAM [27], to systematically identify genes whose expression under hypoxic conditions varied according to cell type. The analysis identified 4,712 Unigene clusters, representing 5,432 array elements, with a FDR of 3.7% (Figure 2A, detailed at: http://microarray-pubs.stanford.edu/hypoxia). A large cluster of genes were induced only in both RPTEC samples (vertical orange bar in Figure 2A; expanded in Figure 2B). A comparison of the number of genes induced and the amplitudes of their inductions in different cell types is shown (Figure 2C). More genes are induced in RPTECs than in HMECs or SMCs. The RPTEC-specific induced genes are involved in many biological processes, including hypoxia metabolic adaptations (HIF-2α), immune regulation (HLA-DRB1, HLA-DRB3, and serine proteinase inhibitor [SERPING1]), solute transport (Multiple Drug Resistance [MDR1], ABCA1, and ABCA5), cell communication (CXCR4), genomic integrity (RAD50, RAD54B, MutS2, MutL3, and AlkB), and several p53 downstream target genes (PTGES, BAI1-associated 3 and proline dehydrogenase, caveolin, and transforming growth factor (TGF)-α (Figure 2B) [34]. Several of the RPTEC-specific hypoxia genes participate in the adaptations to ischemia/perfusion injuries associated with disrupted blood flow to the kidney. SERPING1 is a C1 esterase inhibitor, which regulates complement activation and can protect the cells from ischemia/perfusion injuries [35]. PTGES is a glutathione-dependent prostaglandin E synthase responsible for the production of prostaglandin E2 [36]; prostaglandin E2 is elevated during renal ischemia/perfusion injuries and offers protective functions to the renal tubule cells [36]. Induction of CXCR4, a chemokine receptor for SDF-1, could potentially lead to better tissue repair after tissue injuries due to recruitment of circulating progenitor cells [7]. Interestingly, many of these genes have also been implicated in tumor formation and progression, especially in relation to renal cell carcinoma (RCC). For example, elevated expression of transforming growth factor (TGF-α) in renal cell carcinoma may provide an autocrine stimulation for RCC proliferation [37]; CXCR4 is involved in the progression and metastasis of renal cell carcinoma [38] and breast cancers [39]; and HIF-2α, another inducible member of the HIF family, has been shown to be important for the malignant transformation of renal cell carcinoma [40].

Bi-allelic inactivation of *VHL* genes can cause renal epithelial cells to undergo malignant transformation, leading to renal cell carcinoma [41]. Restoration of a wild-type *VHL* gene into VHL-deficient RCC cells inhibits RCC tumorigenesis [42]. Although the tumor suppression activity of pVHL is thought to be mediated through the degradation of HIF-1α and HIF-2α proteins [40,43], recent evidence suggests the involvement of other oncogenic pathways. We compared the effects of restoration of functional VHL to VHL-mutant RCCs (A498 cells) on global gene expression patterns as reported in a previous study [38] with the RPTEC hypoxia response defined here. Since VHL mediates the degradation of HIF-1α and HIF-2α and thus inhibits the hypoxia pathways, the relative abundance of transcripts in the VHL-mutant A498 cells versus VHL-reconstituted derivatives [38] was compared to the changes in transcript levels of the same set of genes in RPTEC (#1) in response to hypoxia (Figure 2D, detailed at: http://microarray-pubs.stanford.edu/hypoxia). In general, we noted significant similarities between these two biological processes (VHL repression and hypoxia response) with concordant induction (Figure 2D, red vertical bar) and repression (Figure 2D, green vertical bar) of large numbers of genes, including the up-regulation of genes encoding CXCR4, TGF-α, inositol 1,4,5-trisphosphate receptor 1 (ITPR1), REDD1/RTP801, dual specificity phosphatase 1 (DUSP1), cerebroside sulfotransferease (CST), ceruloplasmin (CP), and stanniocalcin (STC1) and the down-regulation of fibronectin 1 (FN), VCAM-1, connective tissue growth factor (CTGF), collagen II (Col II), and aldehyde dehydrogenase 1B1 (ALDH1B1) (Figure 2D) associated with hypoxia stimuli. There were, however, important discrepancies between the responses to these two perturbations. Notable VHL-specific changes were the down-regulation of genes encoding c-MAF, TGF-β, neuregulin, and two TNF-α–induced genes (*TNFAIP3* and *TNFAIP6*) (Figure 2D, vertical black bar). Some of these differences have been noted previously [8] and may be related to cell type-specific features of the response or they may point to hypoxia-independent VHL pathways and VHL-independent hypoxia response pathways.

### Molecular Basis of Strong Hypoxia Responses of RPTECs

**Figure 3:**
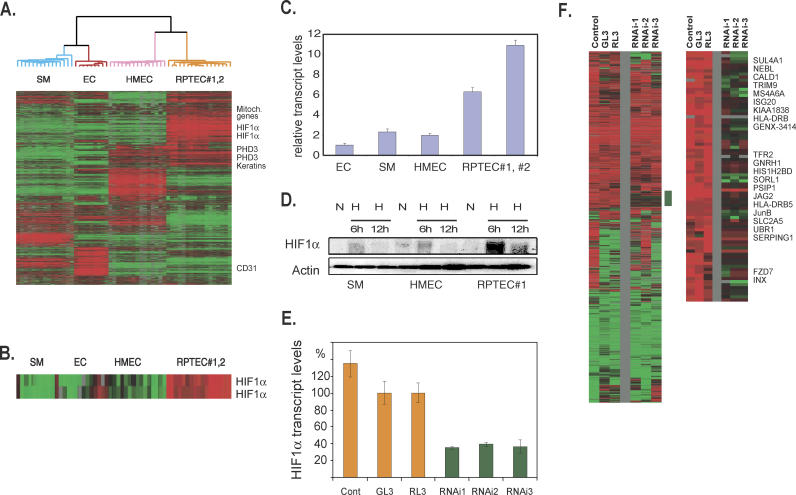
Elevated HIF-1α in RPTECs and Its Role in RPTEC Hypoxia Response (A and B) Hierarchical clustering of a total of 3,630 arrays elements that display a greater than 3-fold change in mRNA expression when placed under hypoxic/anoxic environments in more than four different samples among the 56 samples (A). All samples of the same cell lineages segregate themselves into distinct branches (EC, red; HMEC, pink; RPTEC#1 and #2, orange; SM, blue). The names of some representative genes (mitochondrial genes, *PHD3, CD31,* and genes encoding keratins) are shown to the right, and two elements representing HIF-1α are expanded in (B). (C) The relative expression level of HIF-1α assessed with real-time PCR is shown for different cell types under ambient O_2_ concentration. (D) The levels of HIF-1α protein measured by Western blots of different cells under either ambient air (N) or 2% O_2_ (H) at the indicated times (6 or 12 h). (E) The level of HIF-1α in RPTECs assessed with real-time PCR after the transfection of either control d-siRNAs (transfection fluid alone [Cont] or Dicer-generated siRNAs generated from GL3 or RL3) or d-siRNAs generated from different regions of HIF-1α mRNAs (RNAi-1, 2, and 3). (F) The comparison of hypoxia responses of RPTECs after treatment with either three control or three HIF-1α RNAi transfections. A cluster of genes sensitive to HIF-1α RNAi treatments is marked by a vertical green bar and is expanded with the names of selected genes shown. All the genes with names shown are induced only in RPTECs during hypoxia (Figure 2B). (Complete data can be found at: http://microarray-pubs.stanford.edu/hypoxia)

To investigate the basis of the exceptionally vigorous response to hypoxia observed in RPTECs, we analyzed the global gene expression profiles of all cell types incubated in all oxygen environments without zero transformation. With unsupervised hierarchical clustering, all samples of the same cell type (nine ECs, 15 SMCs, 15 HMECs, and 17 RPTECs [nine RPTEC #1 and 8 RPTEC #2]) cosegregated into four distinct branches (Figure 3A, detailed at: http://microarray-pubs.stanford.edu/hypoxia). Many of the cell type-specific genes were familiar markers of the corresponding cell lineage, such as CD31 (which identifies ECs), and cytokeratins (which are expressed in epithelial cells such as HMECs and RPTECs) (Figure 3A). RPTECs have exceptionally high levels of expression of genes encoded by mitochondrial DNA (Figure 3A). This is consistent with the previous observation that these cells have large numbers of mitochondria, presumably to cope with the high energy demands associated with molecular transport within the renal tubules.

Several genes involved in the regulation of hypoxia response were differentially expressed among different cell types. For example, PHD3/EGLN3, one of the proline hydroxylases that regulates HIF-1α degradation, was more highly expressed in HMECs and RPTECs than in SMCs or ECs (Figure 3A). This immediately suggests the possibility of different mechanisms for oxygen sensing and HIF-1α regulation in these cells. The differential expression of hypoxia response regulators was most clearly evident in the variations in HIF-1α transcripts. The levels of HIF-1α transcripts were higher in all 17 RPTEC samples than in any of the other cells in this study (Figure 3A and 3B), regardless of the oxygen tension during culture. Expression levels of HIF-2α, another member of the HIF family, were similar in all the cell types of this study. Analysis by real-time PCR confirmed that HIF-1α transcript levels were 3- to 5-fold higher in RPTECs than in HMECs or SMCs, while ECs expressed even less HIF-1α mRNA than HMECs or SMCs (Figure 3C). The difference was not due to different culture media—HIF-1α transcripts remained elevated after culturing RPTECs for 24 h in media used for ECs, SMCs, or HMECs. To see whether these elevated HIF-1α transcripts were accompanied by increased protein levels, we analyzed HIF-1α protein levels in SMCs, HMECs, and RPTECs (Figure 3D) under hypoxic conditions (2% O_2_). HIF-1α protein, while undetectable under ambient air, was induced upon exposure to hypoxia in all cells. RPTECs, however, had the highest level of HIF-1α protein induction (Figure 3D), reaching levels that we estimated to be five times higher than those of the other cell types. The RPTECs, then, have the highest levels of HIF-1α at the RNA and protein levels among all tested cells; this characteristic might play an important role in the uniquely strong hypoxia response observed in RPTECs.

To investigate the role that the relatively high HIF-1α levels play in the exaggerated hypoxia response in RPTECs, we used RNAi to reduce the amount of HIF-1α transcripts, and determined the resulting hypoxia response. Since there was no significant preexisting HIF-1α protein when cells were exposed to ambient air, it was not necessary to wait for the degradation of existing HIF-1α proteins after RNAi treatment. To minimize the potential off-target effects of any particular siRNA (J. W. Myers, personal communication) [44,45], we used a mixture of siRNAs generated using in vitro cleavage of the target genes by recombinant Dicer protein, a procedure that results in so-called diced-siRNAs (d-siRNAs) [23]. We generated d-siRNAs from three separate nonoverlapping regions of HIF-1α called HIF-1-α1, 2, and 3 as well as from two controls, GL3 and RL [23]. All three of the HIF-1α-directed d-siRNAs showed an ability to knock down HIF-1α expression while none of the three control siRNA affected HIF-1α transcript levels (Figure 3E) or the induced HIF-1α protein levels during hypoxia (unpublished data). We used DNA microarrays to examine the genomic responses of the d-siRNA-treated RPTECs (using three HIF-1α-directed d-siRNAs or three control-directed d-siRNAs) after 12 h of hypoxia (2%) treatment (Figure 3F, detailed at: http://microarray-pubs.stanford.edu/hypoxia). Although the responses of all the d-siRNA-treated cells were generally similar, we found a cluster of genes whose induction was significantly diminished in all three HIF-1α-RNAi treated RPTEC cultures (Figure 3F, vertical green bar), but not in the three control-RNAi treated cultures. Many of these genes were also induced by hypoxia only in RPTECs and not in the other primary culture cell types we tested (all the genes with names shown in Figure 3F are also present in Figure 2B). Thus, the induction of these RTPEC-specific hypoxia genes appears to be sensitive to the variations in HIF-1α expression, whether the variations were naturally mediated (through cell type-specific expression) or artificially mediated (through the use of d-siRNAs). We conclude that the unusually high level of HIF-1α expression is likely to play a significant role in the exaggerated hypoxia responses observed in RPTECs, and perhaps in their unique vulnerability to transformation when VHL activity is lost.

### The Hypoxia Phenotypes in Human Cancers

Tumor hypoxia is an important factor in human cancer progression [15]. We reasoned that the level of expression of a set of genes characteristically induced by hypoxia in cultured cells might provide a molecular gauge of the presence and extent of the hypoxia response for cancerous human tissues in vivo. Since carcinomas derive from epithelial cells, we selected a cluster of 253 individual elements (representing 168 unique Unigene clusters according to build number 172, released on 17 July 2004) that was consistently induced by hypoxia in cultured epithelial cells and will be referred to as the “epithelial hypoxia signature” (see Figure 1A) for analysis of hypoxia response in vivo. Genes that display the hypoxia signature are generally consistent with independent studies of the global expression response to hypoxia [10,11,14], suggesting that this gene expression signature of hypoxia should provide a good gauge of cellular hypoxia responses.

**Figure 4:**
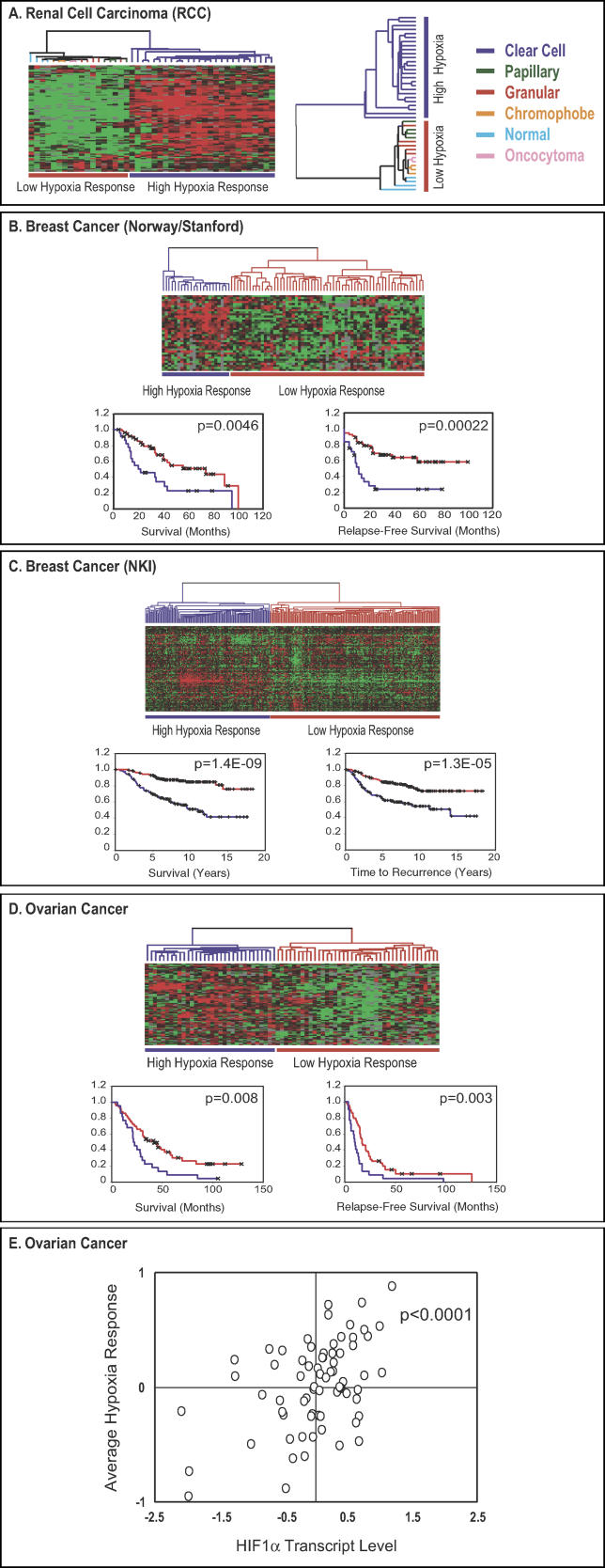
The Analysis of Hypoxia Response in Human Cancers The expression values of genes in the “epithelial cell hypoxia signature” were extracted from an expression study of 41 renal tumors (A) [28]. Genes and samples are organized by hierarchical clustering. The tumors are segregated into two groups defined by high (blue) or low (red) hypoxia response. The histopathological features of renal tumors within the high and low hypoxia response groups are shown. Expression patterns of available “epithelial hypoxia response” genes in a group of breast cancer samples from Norway and Stanford (B) [29] and NKI (C) [47] as well as ovarian cancer samples (D) are shown. The tumors are separated into two groups based on their hypoxia responses, high or low. Kaplan-Meier curves for the clinical outcomes of indicated tumors exhibiting high and low hypoxia responses are shown in (B), (C), and (D). In the Kaplan-Meier curve diagrams, high hypoxia response is indicated by blue, low hypoxia response is indicated by red, and censored patients are indicated with “x” marks. The correlation between the average hypoxia response and HIF-1α RNA levels in 70 ovarian cancer samples is shown in (E).

In a set of global gene expression data from 41 renal tumors [28], we found that the genes within the epithelial hypoxia signature showed remarkable concordance; they were simultaneously elevated in a distinct subset of renal tumors, allowing easy classification of these cancers by hierarchical clustering into two groups, one with high and one with low expression of the hypoxia response genes (Figure 4A, detailed at: http://microarray-pubs.stanford.edu/hypoxia). When we examined the molecular and clinical characteristics of these samples, we found that all the tumors in the “high hypoxia response” group were of the clear-cell RCC type, while the normal kidney samples and tumors of all other histological types were all in the “low hypoxia response” group (Figure 4A). Clear-cell RCCs typically bear a deletion in Chromosome 3p25 that results in the loss of functional VHL proteins [3]. The high hypoxia response signature in clear-cell RCCs is consistent with previous studies that show the activation of hypoxia pathways in these tumors [46], perhaps due to the inappropriate stabilization of HIF-1α and HIF-2α, as a result of the defect in VHL.

We applied a similar approach in examining the hypoxia response in a published breast cancer study [29]. The samples represented four normal breasts, three fibroadenomas, and 78 locally advanced breast cancers with extensive clinical and molecular data. We found that expression of most of the genes in the hypoxia response signature in the breast cancer samples varied in such a way that all the samples were separated into two groups by hierarchical clustering based on the level of hypoxia response (Figure 4B, detailed at: http://microarray-pubs.stanford.edu/hypoxia). All the normal breast samples and fibroadenomas were clustered in a group characterized by generally low expression of the hypoxia response signature, while the ductal adenocarcinoma samples were split between the low and high hypoxia response groups. To investigate whether the two different groups distinguished by levels of hypoxia response might differ in their clinical course, we compared them with respect to overall and relapse-free survival. The patients assigned to the high hypoxia response group had significantly lower overall survival (*p* = 0.0046) and relapse-free survival (*p* = 0.00022) (Figure 4B). Based on analysis of the 69 cancers that had been characterized for the presence or absence of p53 mutations, there was a significant association between high expression of the hypoxia response signature and mutation in p53—18 of 21 samples with high hypoxia response had a p53 mutation as compared to 12 of 48 low hypoxia response samples (*p* = 0.00025, Fisher's exact t-test). The high hypoxia response tumors were also more likely to be negative for estrogen receptor (ER)—10 of 23 high hypoxia response tumors were negative for ER, while only 8 of 51 low hypoxia response tumors were negative (*p* = 0.012)—and more likely to be classified as high-grade—20 of 24 high hypoxia response tumors were high-grade, whereas only 12 of 52 low hypoxia response tumors were high-grade (*p* < 10^−6^) (detailed hypoxia status assignment and their corresponding molecular/clinical data are available at: http://microarray-pubs.stanford.edu/hypoxia).

To assess the consistency and prognostic significance of the hypoxia response signature in an independent set of breast cancer samples, we analyzed a published data set from the NKI, which consisted of 295 early-stage breast cancer samples (stage I and II) [47]. Of the 168 Unigene clusters that defined the epithelial hypoxia response gene cluster in our microarray dataset, 123 genes were also represented (by 176 distinct elements) in the microarrays used in the NKI study. We analyzed variation in expression of these 123 genes, which represent the epithelial hypoxia response signature, in the 295 breast cancer samples. Most of the hypoxia response genes showed consistent variations among tumor samples. Hierarchical clustering, based on similarity in expression of these 123 genes (see Materials and Methods), separated all the tumors into two distinct groups, the high and low hypoxia response groups (Figure 4C, detailed at: http://microarray-pubs.stanford.edu/hypoxia). Because these samples were from patients with early stage disease (stage I and II), the results suggested that significant tumor hypoxia response, or inappropriate activation of a hypoxia response program, could occur even in tumors with diameters of 5 cm or less. Tumors in the high hypoxia response group were associated with much poorer overall survival (*p* = 1.4 × 10^−9^) and higher risk of disease recurrence (*p* = 1.3 × 10^−5^) (Figure 4C), further confirming that the transcriptional signature of a hypoxia response is a significant predictor of poor prognosis for patients with breast cancer.

Using a similar approach, we also examined the hypoxia response of 72 ovarian cancers, all of which are associated with detailed clinical information (MCS, personal communication). The hypoxia signature also showed coordinated regulation and allowed all ovarian cancer samples to be separated into two distinct groups by hierarchical clustering based on hypoxia response (Figure 4D, detailed at: http://microarray-pubs.stanford.edu/hypoxia). These two groups had significant differences in terms of survival (*p* = 0.008) and relapse-free survival (*p* = 0.003) (Figure 4D). Interestingly, in this examination of hypoxia response, HIF-1α levels appeared to co-vary with the level of hypoxia response in different ovarian cancers. We determined the correlation between the HIF-1α and HIF-2α RNA expression levels and the hypoxia responses of individual ovarian tumors by converting numerical gene expression values to a logarithmic scale and averaging them. We have found a significant correlation between the average hypoxia responses and HIF-1α transcript levels (*p* < 0.0001, Figure 4E) but not with HIF-2α transcript levels (*p* = 0.6121). These data suggest that variations in the HIF-1α transcript levels may play a role in determining the degree of the hypoxia response of ovarian tumors as it does in the vigorous hypoxia response observed in RPTECs.

**Figure 5:**
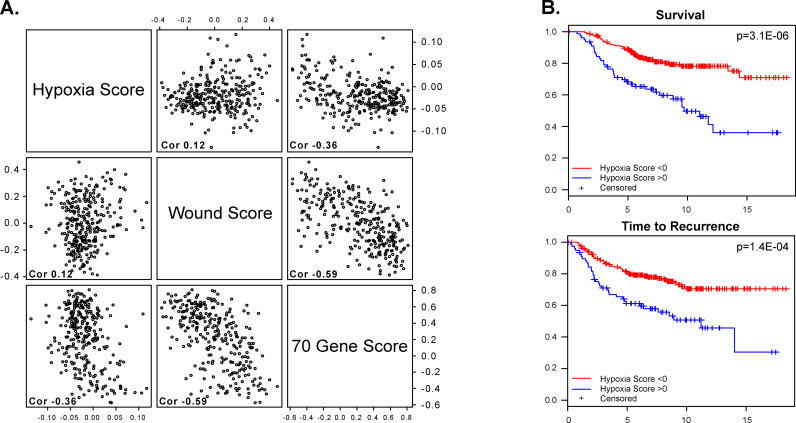
Quantitative Analyses of the Prognostic Significance of the Hypoxia Response Signature Response signature was analyzed in the 295 breast cancer samples in the NKI study (A) Scatter plots showing the relationship between the value of the average expression level of the genes in the hypoxia signature and that of genes in the wound response signature [22] or 70-gene signature [48]. Each point in the scatter plots represents a single one of the 295 tumors analyzed in the NKI dataset. The overall correlation between each pair of expression signatures across this set of 295 samples is indicated in each panel. (B) With the threshold value of the hypoxia response signature for classification of patients into high and low hypoxia response groups set at zero, the Kaplan-Meier curve shows significant differences in survival and time to recurrence between the samples in the high and low hypoxia response groups of these breast cancer samples.

To provide a metric that can be prospectively applied to new tumor samples, we defined a quantitative “hypoxia response score” for each sample by simply averaging the gene expression levels (converted to logarithmic scale) for the 123 genes of the hypoxia response signature. Setting this metric also allowed us to investigate the relationship between the hypoxia signature and other previously identified prognostic gene expression signatures. One such prognostic signature was based on expression of a set of 70 genes that was first identified in a “supervised” fashion based on their ability to predict freedom from tumor metastasis (favorable prognosis) over a five-year period in the same dataset [48]. A second signature, related to a wound response signature, was identified in a study of fibroblast exposure to serum and shown to predict risks of progression and metastasis of breast cancer [22]. In these tumors, the hypoxia response score was only very weakly correlated with the wound response score (correlation = 0.12), or with the 70-gene score (correlation = −0.36). The wound response score, on the other hand, showed a strong (negative) correlation with the 70-gene score (correlation = −0.59) (Figure 5A).

The hypoxia response score could be further simplified by thresholding its value at 0.0; patients with a score greater than zero are classified in the “high hypoxia response” group, and those less than zero the “low hypoxia response” group. When we compared the clinical outcomes of patients in these two different groups, we found that patients in the high hypoxia response group do much worse in terms of survival (*p* = 3.1 × 10^−6^) and the risk of disease recurrence (*p* = 1.4 × 10^−4^) than the patients in the low hypoxia response group (Figure 5B).

While thresholding the score at zero produced a striking classification, this cutoff point was somewhat arbitrary. To investigate whether an alternative threshold might improve predictive power, we refit our multivariate Cox model by including the hypoxia response score in a quantitative form, using natural cubic splines with four degrees of freedom. These curves estimated the differential contribution of the hypoxia response score to the (log) relative risk in a continuous fashion (see Figure S1). The results showed a strong positive trend over the range of hypoxia response scores in which most of the data occur, and indicate that a threshold value of zero was a reasonable choice.

### Prognostic Information Content of the Hypoxia Signature

We wanted to know whether the hypoxia signature adds anything new and useful to clinical decision-making and whether it significantly complements previously established prognostic factors. To this end, we included the hypoxia response score in a multivariate Cox model, along with other established prognostic and clinical factors (including the wound response signature [22]). Remarkably, the hypoxia signature contributed more significantly to the predictive power of the model than did chemotherapy, ER status, tumor size, grade, angioinvasion, or age; its contribution was comparable to that of the wound signature but less than the NKI 70-gene signature (Tables 1, 2, S1, and S2). This further confirms the importance of the hypoxia response signature as an independent predictor of poor prognosis. The hypoxia response score was significantly associated with both survival (*p* = 0.003; relative risk = 2.387; 95% confidence interval, 1.357–4.198) and time to recurrence (*p* = 0.004; relative risk = 2.164; 95% confidence interval, 1.284–3.647) (Table 1). We also calculated the contribution of individual factors in a multivariate Cox model by assessing the differences in predictive power of the model with or without any individual factors. Adding the hypoxia response signature to a Cox model for survival that included the wound response signature and established prognostic factors accounted for an additional 9.4% of the variance in survival. This contribution was more than the variance accounted for by tumor grade, diameter, or angioinvasion, similar to the contribution of ER status, but less than the variation explained by age or the wound response signature. The hypoxia response signature and wound response signature together accounted for 40.8% of the prognostic power of the multivariate models (analysis detailed in data file Cox_factor_model at: http://microarray-pubs.stanford.edu/hypoxia).

**Table 1:**
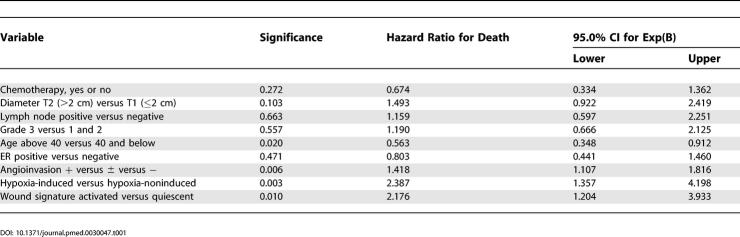
Epithelial Hypoxia-Induced, Wound Signature, and Clinical Risk Factors: Survival (Cox Regression)

**Table 2:**
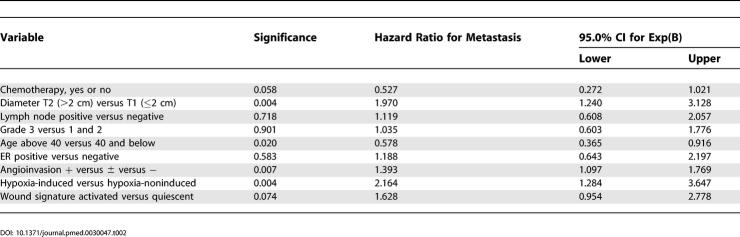
Epithelial Hypoxia-Induced, Wound Signature, and Clinical Risk Factors: Metastasis as First Event (Cox Regression)

## Discussion

In this study, we first examined the diversity of gene expression responses to hypoxia among cultured cells of different origins, and found significant differences in the amplitude and breadth of the responses. Both of the epithelial cell types (HMECs and RPTECs) we tested showed a higher amplitude of gene induction than the two stromal cells (ECs and SMCs). Although the root of this observation is not yet clear, it is consistent with previous studies in which most of the hypoxia-induced genes (e.g., *GLUT-1* and *CA IX*) in cancerous tissues were identified preferentially in carcinoma cells but not in the adjacent stromal cells [49], although both cell types are presumably exposed to similar oxygen microenvironments. RPTECs have especially vigorous transcriptional responses to hypoxia, which appear to be due, at least partially, to the high levels of HIF-1α expression in these cells. In support of this idea, we found that RNAi-mediated knock-down of HIF-1α expression greatly diminishes the induction of many RPTEC-specific hypoxia response genes. Furthermore, the positive correlation between the HIF-1α RNA level and the amplitude of the hypoxia response in ovarian cancer suggests that the transcriptional control of HIF-1α may also play a role in the ovarian cancer hypoxia responses. It is likely that this cellular fine-tuning of the magnitude of the hypoxia response or sensitivity to hypoxia may be achieved by regulating the abundance and composition of the molecular machinery involved in the hypoxia response. There are undoubtedly many more variations in the hypoxia responses still to be discovered and explored in other cell types. Understanding the molecular system that determines these differences will be critical to dissecting the physiological and pathological responses to variations in oxygen availability at the tissue and organism levels [50].

Patients with von Hippel-Lindau disease, who have a defective copy of the gene encoding VHL protein, are at remarkably increased risk of cancer, with the majority of epithelial cancers in these patients being clear-cell renal cell carcinomas. This specific type of RCC is thought to originate from RPTECs [3]. But why are RPTECs preferentially affected, although the VHL mutation is present in all cell types? The uninhibited hypoxia response is thought to underlie the oncogenic transformation associated with the loss of VHL, because inhibition of HIF activities [40,43] can halt the malignant transformation and aggressive behavior of VHL-deficient RCCs. The unusually high levels of HIF-1α expression and strong hypoxia response in normal RPTECs might make them especially vulnerable to a defect in the degradation of HIF protein in von Hippel-Lindau disease. Many of the RPTEC-specific transcriptional changes in response to hypoxia have been suggested to contribute to the pathogenesis of clear-cell RCCs (through the actions of HIF-2α, TGF-α, CXCR4, and MDR1). Although the exact nature of the exceptionally vigorous hypoxia response of RPTECs, and its contribution to the formation of clear-cell RCC is still to be determined, our results raise the possibility that inhibiting the systems underlying the vigorous hypoxia response of RPTECs might have utility in inhibiting the development of RCC in von Hippel-Lindau disease.

In this study, we were also able to stratify human cancers according to the presence and amplitude of a hypoxia response, as reflected in a gene expression signature that we defined in a cell culture model. In breast and ovarian cancers, tumors with a strong gene expression signature of the hypoxia response had a significantly worse prognosis. The correlation of the hypoxia response signature with cancer progression and metastasis immediately raises the question of whether the hypoxia response actually contributes to the enhanced aggressiveness of these cancers and their poor therapeutic response. Treatment resistance of hypoxic tumors has been ascribed to factors in tumor microenvironments, such as failure of local drug delivery during chemotherapy or inability to generate free oxygen radicals during radiation therapy [15]. However, many in vitro studies have revealed that hypoxia can alter cell properties to directly contribute to tumor development, progression, and drug resistance through various mechanisms, such as up-regulation of MDR1 (drug resistance) [51], telomerase (cell immortality) [52,53], CXCR4 expression (cell migration and metastasis) [38], and down-regulation of the DNA repair system (genomic instability) [54]. These data suggest that the hypoxia-induced gene expression program may have a direct causal role in impacting the tumor biology to affect clinical outcomes. It will be important, then, to understand what specific hypoxia-driven biological processes underlie the phenotypic differences between the tumors in the high-hypoxia response group and those in the low-hypoxia response group.

The mechanisms underlying the variation in hypoxia responses in breast and ovarian tumors are still unknown. There are three reasonable possibilities; variations in the hypoxia response program could reflect: (1) actual variations in oxygen tension in the tumors; (2) cell type-specific variations in the magnitude of, or threshold for, the response to bona fide hypoxia, similar to those seen in our analysis of different normal cells; or (3) inappropriate activation of the hypoxia response resulting from genetic and/or epigenetic alterations in cancers. Although the hypoxia response in tumors is usually thought to be caused by the first mechanism, the evidence suggests contributions from the second and third mechanisms as well. For example, activation of the hypoxia response program in clear-cell RCC is almost certainly caused by loss of VHL (see Figure 4A) [4] rather than by low oxygen tension. In breast cancer, the over-representation of p53 loss-of-function mutations in the tumors with elevated hypoxia responses suggests that the loss of the p53′s role in inhibiting HIF-1α protein stability and hypoxia-induced cell death [4,55–57] may be a factor in these tumors. Other oncogenic alterations in regulatory systems [3–7] might also play a role in triggering or modifying the hypoxia response in human cancers; a dissection of the contributions of tumor oxygen levels and disordered regulation of the hypoxia response in individual tumors will, therefore, be important in developing therapeutic strategies based on exploitation or inhibition of this program.

By using systematic analysis of global gene expression patterns to relate in vitro cell culture models to their in vivo cancer counterparts, we have been able to identify gene expression patterns characteristic of two specific regulatory programs: a wound response [22], and a hypoxia response (this study) that together account for more than 40% of the predictive power of a multivariable prognostic model that includes all the classical prognostic factors for breast cancer survival. These results show the power of controlled ex vivo studies in defining gene signatures that allow the recognition of critical molecular programs in cancers. The results also show the potential for improvement in the current criteria for risk stratification of cancer patients. The prognostic information in the hypoxia signature was virtually independent of that provided by the previously reported wound signature [22] and a 70-gene prognostic score [48]. Thus, integrating information representing diverse genetic and molecular characteristics with clinical data, to build a detailed profile of the biology of each tumor, can greatly improve our ability to predict its clinical course and choose the appropriate therapeutic strategy [58]. Unlike the “top-down” supervised approach to identifying gene expression-based predictive models of cancer outcomes, in which no specific biological processes are associated with the tumor phenotypes, this “bottom-up” approach directly links cancer phenotypes to specific molecular programs. Equally importantly, it links prognostic molecular signatures to ex vivo experimental models that can be used to study them, providing a more direct route to the development of targeted therapeutics [59]. Many therapeutic agents are already under development to specifically target HIF pathways [2], or to target cells under hypoxic environments by hypoxia-selective cytotoxins and hypoxia-dependent gene therapy [60,61]; these drugs may offer the greatest benefits to cancer patients with a high hypoxia response.

## Supporting Information

Figure S1 Quantitative Measurement of Hypoxia Score and Corresponding Clinical Risks The correlation of the quantitative and continuous hypoxia response score (x-axis) of each tumor sample (black circle) in the NKI breast cancer dataset [48] versus the (log) relative risk (y-axis) with in terms of survival and metastasis-free survival.
(153 KB PDF).

Table S1 Multivariate Analysis of Risk Factors Using Classical Clinical Predictors Alone Multivariate analysis of risk factors for death (A) and metastasis (B) as the first recurrence event in early breast cancer using a predictive model containing only classical clinical predictors.
(37 KB PDF).

Table S2 Multivariate Analysis of Risk Factors Using Classical Clinical Predictors Combined with Hypoxia Signature and 70 Gene Predictors Multivariate analysis of risk factors for death (A) and metastasis (B) as the first recurrence event in early breast cancer using a predictive model containing several classical clinical predictors as well as the gene signature of hypoxia response and NKI 70-gene predictor [48].
(42 KB PDF).

### Accession Numbers

Our microarray experiment data were deposited in the GEO database (http://www.ncbi.nlm.nih.gov/geo) and assigned the accession number GSE3537.

**Patient Summary**

**Background**

All human cells need oxygen and have elaborate mechanisms to sense inadequate oxygen levels and to respond in ways that minimize damage. Low oxygen conditions, also called hypoxia, are characteristic of many tumors. Several previous studies have suggested that tumors that are more starved of oxygen (more hypoxic) are also more aggressive and associated with a poor prognosis for the patient.

**Why Was This Study Done?**

The researchers who did this study wanted to thoroughly characterize the response of different types of cells to hypoxia. They also wanted to see whether a tumor's response to hypoxia held information that could help doctors decide about the best treatment.

**What Did the Researchers Do and Find?**

They used a type of genetic tool called DNA microarrays (these can measure changes in activity across thousands of genes) to examine the response to hypoxia in a number of different cell types. There are some differences in how individual cell types (kidney cells, breast cells, or muscle cells, for example) respond to hypoxia, but also many similarities. From the changes in gene activity that were common to different cell types, the researchers selected a representative group, which they called the “hypoxia response signature.” They then examined the hypoxia response signature in several human cancer types, including kidney, breast, and ovarian cancer. They found that, consistently, patients with tumors whose gene activities were similar to the hypoxia signature were worse off. They were more likely to have a recurrence of the cancer after surgery and less likely to survive for five years or longer after the initial cancer diagnosis. The researchers also found that the results from the hypoxia signature test provided more information about the course of a patient's illness than any one of the clinical parameters that doctors take into account when making decisions on how to treat a particular cancer patient (such as tumor size and grade, patient age, and whether a breast cancer is estrogen-responsive or not).

**What Does This Mean?**

The study suggests that measuring how closely a tumor's gene activity pattern matches the hypoxia signature yields information that is relevant for clinical decision-making. Much more work is necessary before it is clear whether such a test is useful and practical in normal clinical settings. However, like a number of other recent studies, this one suggests that applying new molecular technologies can reveal important differences between tumors that can't be seen by other means but are relevant for the best choice of treatment.

**Where Can I Find More Information Online?**

The following Web sites contain general information about cancer.

Pages from the American Cancer Society (search for microarray): http://www.cancer.org/docroot/home/index.asp

Pages from Cancer Research UK:

http://www.cancerresearchuk.org/

Pages from the US National Cancer Institute:

http://www.cancer.gov/

MedlinePLus pages on cancer:

http://www.nlm.nih.gov/medlineplus/cancer.html

## Abbreviations

d-siRNA: diced-siRNA
EC: endothelial cell
ER: estrogen receptor
FDR: false discovery rate
GL3: firefly luciferase
HIF: hypoxia-inducible factor
HMEC: human mammary epithelial cell
NKI: Netherlands Cancer Institute
PHD: prolyl hydroxylase
pVHL: von Hippel-Lindau protein
RCC: renal cell carcinoma
RL: Renilla luciferase
RNAi: RNA interference
RPTEC: renal proximal tubule epithelial cell
SAM: significance analysis of microarrays
siRNA: small interfering RNA
SMC: smooth muscle cell
TGF: transforming growth factor
