# Supplementary material for: Gene Expression Programs in Response to Hypoxia: Cell Type Specificity and Prognostic Significance in Human Cancers
Source: PLoS Med. 2006 Jan 24;3(3):e47. doi: 10.1371/journal.pmed.0030047 (PMC1334226; doi:10.1371/journal.pmed.0030047)
Supplement: Supplementary file 1 [file pmed.0030047.sg001.pdf]

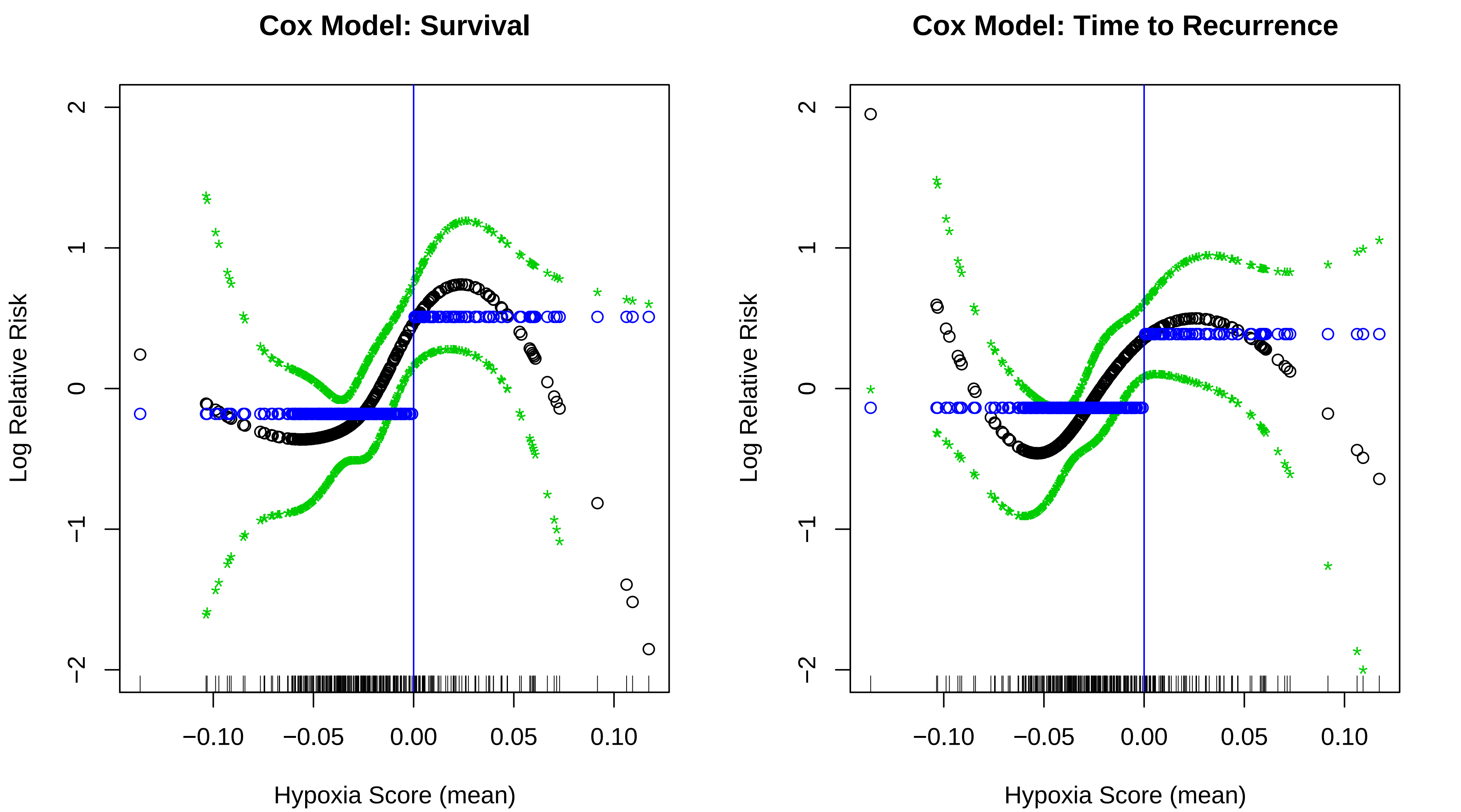

Cox Model: Survival
Log Relative Risk
2
1
0
−1
−2
−0.10
−0.05
0.00
0.05
0.10
Hypoxia Score (mean)
Cox Model: Time to Recurrence
Log Relative Risk
2
1
0
−1
−2
−0.10
−0.05
0.00
0.05
0.10
Hypoxia Score (mean)
