# Supplementary material for: Gene Expression Programs in Response to Hypoxia: Cell Type Specificity and Prognostic Significance in Human Cancers
Source: PLoS Med. 2006 Jan 24;3(3):e47. doi: 10.1371/journal.pmed.0030047 (PMC1334226; doi:10.1371/journal.pmed.0030047)
Supplement: Supplementary file 2 [file pmed.0030047.st001.pdf]

Supplemental Table S1

Survival – Clinical Variable only

	Significance	Hazard Ratio	95.0% CI for Hazard Ratio	
		For Death	Lower	Upper
Diameter T2 (> 2cm) vs. T1 (=< 2cm)	0.041	1.653	1.020	2.679
Lymph node positive vs. negative	0.407	1.342	0.669	2.694
Grade III vs. II vs. I	0.022	1.865	1.094	3.180
Age above 40 vs. 40 and below	0.031	0.583	0.357	0.951
ER positive vs. negative	0.0004	0.408	0.249	0.671
Angio invasion + vs.+/-, vs. -	0.002	1.458	1.142	1.860
Chemotherapy Yes versus No	0.310	0.691	0.338	1.411
Hormonal therapy Yes versus No	0.686	0.831	0.338	2.040

Distant Metastasis as First Event – Clinical Variable only

	Significance	Hazard Ratio	95.0% CI for Hazard Ratio	
		For Metastasis	Lower	Upper
Diameter T2 (> 2cm) vs. T1 (=< 2cm)	0.001	2.152	1.360	3.407
Lymph node positive vs. negative	0.326	1.374	0.728	2.592
Grade III vs. II vs. I	0.166	1.414	0.866	2.309
Age above 40 vs. 40 and below	0.039	0.614	0.386	0.976
ER positive vs. negative	0.123	0.669	0.402	1.115
Angio invasion + vs.+/-, vs. -	0.003	1.419	1.123	1.795
Chemotherapy Yes versus No	0.049	0.509	0.260	0.997
Hormonal therapy Yes versus No	0.332	0.664	0.291	1.517
