# Supplementary material for: Gene Expression Programs in Response to Hypoxia: Cell Type Specificity and Prognostic Significance in Human Cancers
Source: PLoS Med. 2006 Jan 24;3(3):e47. doi: 10.1371/journal.pmed.0030047 (PMC1334226; doi:10.1371/journal.pmed.0030047)
Supplement: Supplementary file 3 [file pmed.0030047.st002.pdf]

Supplemental Table S2

Survival – Clinical Variable, Hypoxia and 70-genes Prognosis Profile

	Significance	Hazard Ratio	95.0% CI for Hazard Ratio	
		For Death	Lower	Upper
Diameter T2 (> 2cm) vs. T1 (=< 2cm)	0.026	1.733	1.069	2.809
Lymph node positive vs. negative	0.324	1.420	0.708	2.846
Grade III vs. II vs. I	0.963	1.013	0.582	1.765
Age above 40 vs. 40 and below	0.032	0.581	0.354	0.954
ER positive vs. negative	0.295	0.755	0.446	1.277
Angio invasion + vs.+/-, vs. -	0.014	1.362	1.064	1.743
Chemotherapy Yes versus No	0.174	0.603	0.291	1.250
Hormonal therapy Yes versus No	0.481	0.718	0.287	1.801
Hypoxia (Hypoxic vs Non-Hypoxic)	0.001	2.494	1.434	4.336
70 genes (poor vs good)	0.0002	4.987	2.162	11.507

Distant Metastasis as First Event – Clinical Variable, Hypoxia and 70-genes Prognosis Profile

	Significance	Hazard Ratio	95.0% CI for Hazard Ratio	
		For Death	Lower	Upper
Diameter T2 (> 2cm) vs. T1 (=< 2cm)	0.001	2.190	1.383	3.467
Lymph node positive vs. negative	0.401	1.313	0.695	2.479
Grade III vs. II vs. I	0.453	0.822	0.494	1.370
Age above 40 vs. 40 and below	0.095	0.668	0.416	1.073
ER positive vs. negative	0.630	1.143	0.664	1.967
Angio invasion + vs.+/-, vs. -	0.024	1.318	1.037	1.675
Chemotherapy Yes versus No	0.034	0.479	0.242	0.945
Hormonal therapy Yes versus No	0.219	0.590	0.255	1.367
Hypoxia (Hypoxic vs Non-Hypoxic)	0.012	1.888	1.149	3.102
70 genes (poor vs good)	<0.0001	4.043	2.117	7.721
